# Uveal Melanoma Identified as Ocular Mass on Point-of-care Ultrasound

**DOI:** 10.5811/cpcem.2021.4.52115

**Published:** 2021-07-27

**Authors:** Hannah Spungen, Daniel Weingrow

**Affiliations:** University of California, Los Angeles, Department of Emergency Medicine, Los Angeles, California

**Keywords:** Vision loss, uveal melanoma, point-of-care ultrasound

## Abstract

**Case Presentation:**

A 41-year-old man presented to the emergency department with five months of progressive monocular vision loss in his right eye, which he described as a gradually descending and enlarging black spot. He had no light perception in his right eye with elevated intraocular pressure and an afferent pupillary defect, while his left eye visual acuity and pupillary exam was normal. Point-of-care ultrasound demonstrated a hyperechoic, pedunculated mass in the posterior chamber of his right eye, consistent with a diagnosis of ocular melanoma. Ophthalmology scheduled the patient for an elective, right eye enucleation the following week, after which a diagnosis of uveal melanoma (UM) was confirmed on histopathology.

**Discussion:**

Uveal melanoma is an uncommon diagnosis that requires prompt intervention and surveillance due to the possibility of distant metastases arising in up to 50% of patients. Emergency department diagnosis of UM may be confounded by features of other intraocular pathology, such as increased ocular pressure or the finding of retinal detachment on fundoscopy. When emergency providers encounter glaucoma or retinal detachment on physical exam, point-of-care ultrasonography represents a key adjunct in the timely diagnosis and referral of this potentially vision- and life-threatening malignancy.

## CASE PRESENTATION

A 41-year-old male with no known medical history presented to the emergency department (ED) with five months of progressive vision loss in his right eye. He initially noticed a black area over the superior hemifield of his right eye that progressed downward in a curtain-like fashion over the succeeding months. There was no associated eye pain or headache. On physical exam, he was noted to have no light perception in his right eye and 20/20 visual acuity in the left. Confrontational visual field testing in the right eye showed a complete visual field cut. His right pupil was 6 millimeters (mm) and sluggish with an afferent pupillary defect, while the left pupil was 5 mm and briskly reactive. Intraocular pressures were 40 millimeters mercury (mm Hg) and 14 mm Hg in the right and left eyes, respectively (reference range 10–20 mm Hg). His cranial nerve exam was unremarkable except for the ocular components described above, and strength and sensory testing was normal in the bilateral upper and lower extremities. A point-of-care ocular ultrasound revealed a pedunculated, hyperechoic mass in the posterior chamber (Image).

Ophthalmology was consulted in the ED and scheduled the patient for right eye enucleation the following week, at which time histopathology confirmed the diagnosis of uveal melanoma (UM). Surveillance imaging for primary metastases was recommended at the time of postoperative discharge.

## DISCUSSION

Intraocular masses such as UM are traditionally diagnosed using fundoscopy. Uveal melanoma has a stereotypical appearance on ultrasound described as a mushroom or pedunculated shape with regular internal structure and internal vascularity.^1^ Patients may present with visual disturbances or vision loss, although approximately 30% are discovered incidentally.^2^ Increased intraocular pressures and angle-closure glaucoma have also been reported in UM from mass effect-related compressive or rotational angle closure.^1^ Prompt diagnosis of UM is clinically important because up to 50% of cases go on to develop distant metastases,^3^ by which time mean survival drops to 6–12 months.^4^ In patients with unilateral features such as ocular pain, vision loss, or elevated intraocular pressure, bedside ultrasonography plays a vital role in the diagnosis of intraocular masses.

**CPC-EM Capsule**

What do we already know about this clinical entity?

*Uveal melanoma is a rare but potentially lethal condition that may present as subacute monocular vision loss with possible unilateral increase in intraocular pressure*.

What is the major impact of the image(s)?

*The stereotypical pedunculated, mushroom-like appearance of uveal melanoma is clearly demonstrated*.

How might this improve emergency medicine practice?

*This ultrasound finding should raise the practitioner’s suspicion for malignancy and prompt timely ophthalmologic and possibly oncologic referrals*.

## Supplementary Information

Video Ultrasound clip showing intraocular lens dislocation (arrow) into the posterior chamber. The prosthetic lens appears as a hyperechoic curvilinear structure in the posterior chamber with the temporal side haptic still adherent to the lens capsule, whereas an appropriately positioned prosthetic lens would appear within the lens capsule posterior to the iris.

## Figures and Tables

**Image:**
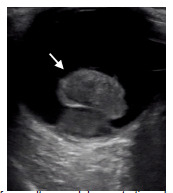
Point-of-care ultrasound demonstrating a hyperechoic, pedunculated mass (white arrow) in the posterior chamber of the patient’s right eye, consistent with a diagnosis of uveal melanoma.
